# Blood T_1_ measurements using slice-interleaved T_1_ mapping (STONE) sequence

**DOI:** 10.1186/1532-429X-18-S1-P57

**Published:** 2016-01-27

**Authors:** Steven Bellm, Long Ngo, Jihye Jang, Sophie Berg, Kraig V Kissinger, Beth Goddu, Warren J Manning, Reza Nezafat

**Affiliations:** 1 grid.38142.3c000000041936754XMedicine, Beth Israel Deaconess Medical Center, Harvard Medical School, Boston, MA USA; 2 grid.38142.3c000000041936754XRadiology, Beth Israel Deaconess Medical Center, Harvard Medical School, Boston, MA USA

## Background

Slice interleaved T_1_ (STONE) mapping sequence was recently proposed to take advantage of increased recovery time of spins to improve accuracy and precision of native myocardial T_1_ values. In this sequence, a non-selective inversion pulse is followed by acquisition of the data for different slices. Therefore, blood pool may experience different recovery time at different slice location due to mixing effect and rapid blood flow movement. This may impact ECV measurements using STONE based T_1_ mapping sequence. While, a short T1 of blood after contrast allows full recovery, long native T1 of blood pool may cause errors in T_1_ measurements, which will manifest as low reproducibility and variations across different locations. Therefore, we sought to assess the native blood T_1_ values measured in the right ventricle (RV) and left ventricle (LV) by studying the reproducibility of T_1_ measurements at different locations and slices.

## Methods

Nine healthy subjects (38 ± 22 years, 4 males) were recruited to participate in an IRB-approved study for imaging. Each subject was in sinus rhythm and was imaged 5 times using STONE sequence with gradient echo readout (STONE-GRE) and steady-state free precession sequence (STONE-SSFP). T_1_ maps were reconstructed after motion correction and voxel-wise curve fitting using a 2-paramter fit model. The region of interest (ROI) for the blood pool was manually marked on five short-axis slices for RV as well as for LV to generate slice-based native T_1_ values of the blood pool. Coefficient of variation (CV) analysis for each sequence was used to assess the variability of T_1_ measurements between each slice and between the repetitions of measurements.

## Results

**Figure 1:**
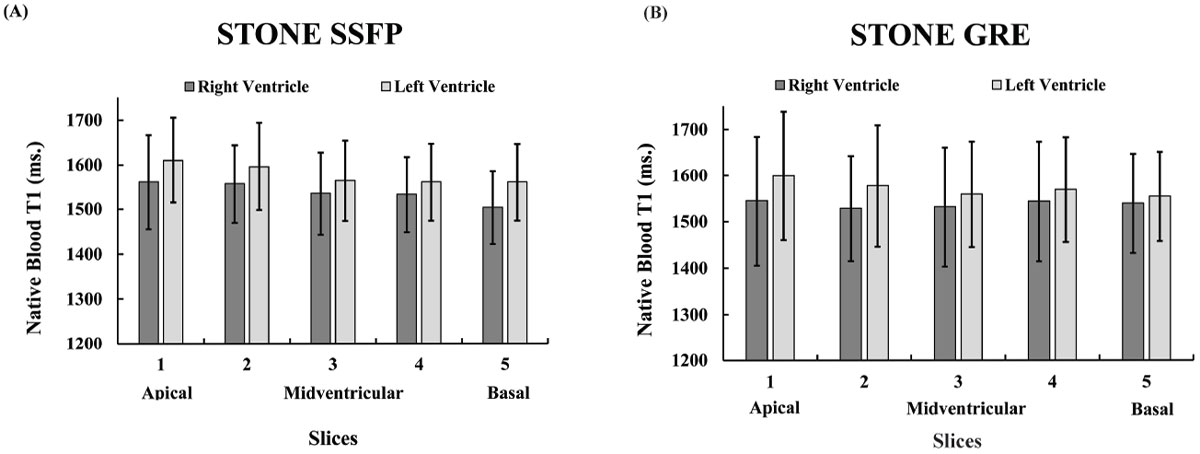
**Mean native T1 of the blood pool per 5 slices in STONE SSFP (A) and STONE GRE (B) for RV and LV**.

**Figure 2:**
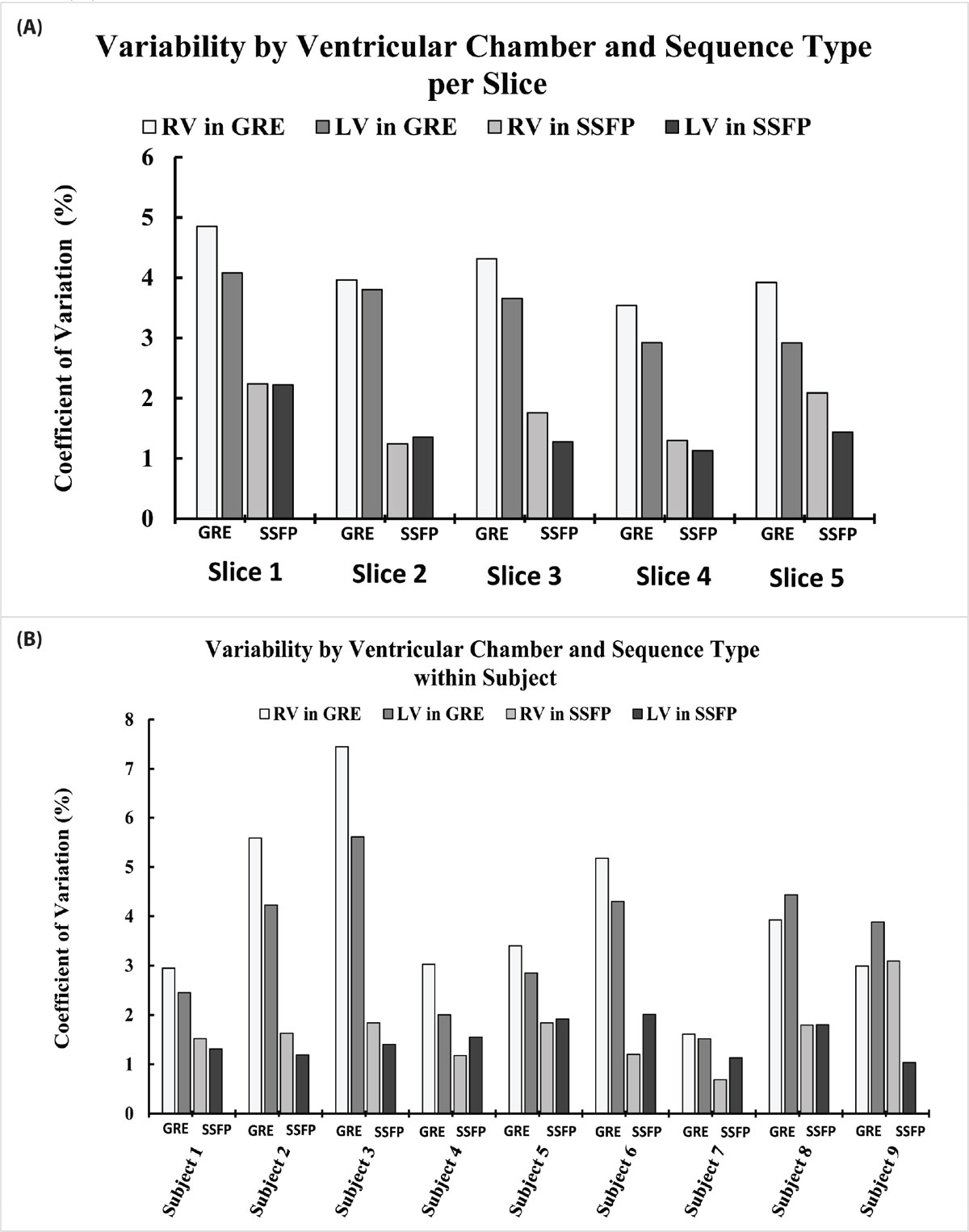
**Variability of native T1 measurements of the blood pool by ventricular chamber and sequence type within each slice (A) and by ventricular chamber and sequence type within each subject (B)**.

Figure 1 shows mean T_1_ values averaged over all subjects for STONE-SSFP and STONE-GRE. T_1_ means in RV were systematically smaller than in LV for both sequences (p < 0.05). Figure 2 (A) shows a high reproducibility (GRE: 3.8 ± 0.6%, SSFP: 1.6 ± 0.4%) among 5 slices with significantly smaller CVs in STONE SSFP than in GRE (p< 0.05). There was similar variability among the 5 slices for each sequence. Figure 2 (B) shows variability among the subjects with higher reproducibility for STONE SSFP (GRE: 3.7 ± 1.5%, SSFP: 1.6 ± 0.5%).

## Conclusions

Native blood T_1_ measurements with STONE sequence are reproducible in both LV and RV and there is no systematic difference in T_1_ measurements at difference slice locations within LV or RV. However, there are differences in T_1_ measurements of LV vs. RV for both sequences.

